# Understanding the physicochemical properties and degradation kinetics of nicotinamide riboside, a promising vitamin B_3_nutritional supplement

**DOI:** 10.29219/fnr.v63.3419

**Published:** 2019-11-21

**Authors:** Michael T.D. Campbell, David S. Jones, Gavin P. Andrews, Shu Li

**Affiliations:** School of Pharmacy, Queens University Belfast, Belfast, Norther Ireland, UK

**Keywords:** nicotinamide riboside, nutritional supplementation, physicochemical properties, degradation kinetics, in vitro testing

## Abstract

Nicotinamide riboside (NR), a newly recognised form of vitamin B_3_ and a precursor to nicotinamide adenine dinucleotide (NAD^+^), has been demonstrated to show therapeutic potential and the possibility of becoming a drug compound in addition to its proven role in rejuvenating ageing cells in mice. However, current literature is devoid of information relating to the physicochemical characterisation of NR and its respective impact upon formulation and final product processing. Here we report physicochemical properties of NR including p*K*a, log *P*, solubility, melting point, degradation mechanics, and kinetics, with a special focus on its stability under thermal and physiologically relevant conditions. A simple and rapid HPLC method confirms a base-catalysed hydrolysis degradation of NRCl to nicotinamide and sugar in simulated gastrointestinal (GI) fluids. Given the antagonising effect of nicotinamide against NR, the presented data have a profound impact on how NRCl should be handled both during formulation and storage to prevent formation and to limit accumulation of nicotinamide. The innovative combinatorial use of ^1^H NMR and Differential Scanning Calorimetry (DSC) was employed to investigate thermal events during NR melting. NRCl degrades upon melting and in solution undergoes hydrolysis in a buffer and in simulated intestinal environments. The results suggest that a proper and evidence-based formulation of NRCl is vital to enable further investigation and clinical analysis of this promising and novel nutrient. Any formulation would need to promote the stability of NRCl and protect it from hostile environments to prevent the accumulation of a potentially antagonistic degradation product. With the current work, we have filled a niche but vital gap in NR literature and the data presented may prove useful in furthering the understanding, specifically the formulation and processing of NRCl.

## Popular scientific summary

- Nicotinamide riboside (NR) has gained increasing attention over the last decade as a newly developed and promising nicotinamide adenine dinucleotide (NAD+) precursor.
- A crystalline NR form (NRCl in a capsulated form called NIAGEN) has been used in numerous clinical studies to understand the role of NR and its NAD+ boosting effect on pathologies for a number of age-related diseases.
- Seldom effort, however, has been made to understand formulation and manufacturing requirements of this novel molecule.
- In this paper, key physicochemical properties of NR including pKa, log P, solubility, melting point, degradation mechanics, and kinetics, with a special focus on its stability under thermal and physiologically relevant conditions, were investigated.
- The results have shown that NRCl is a labile molecule that degrades to form a possibly antagonistic product when exposed to either gastric fluid or elevated temperature.
- Therefore, careful formulation design, and rational processing means/parameter selection are vital in early development of NRCl dosage forms to ensure successful delivery of an accurate dose for optimised physiological effects.

**Fig. 1:**
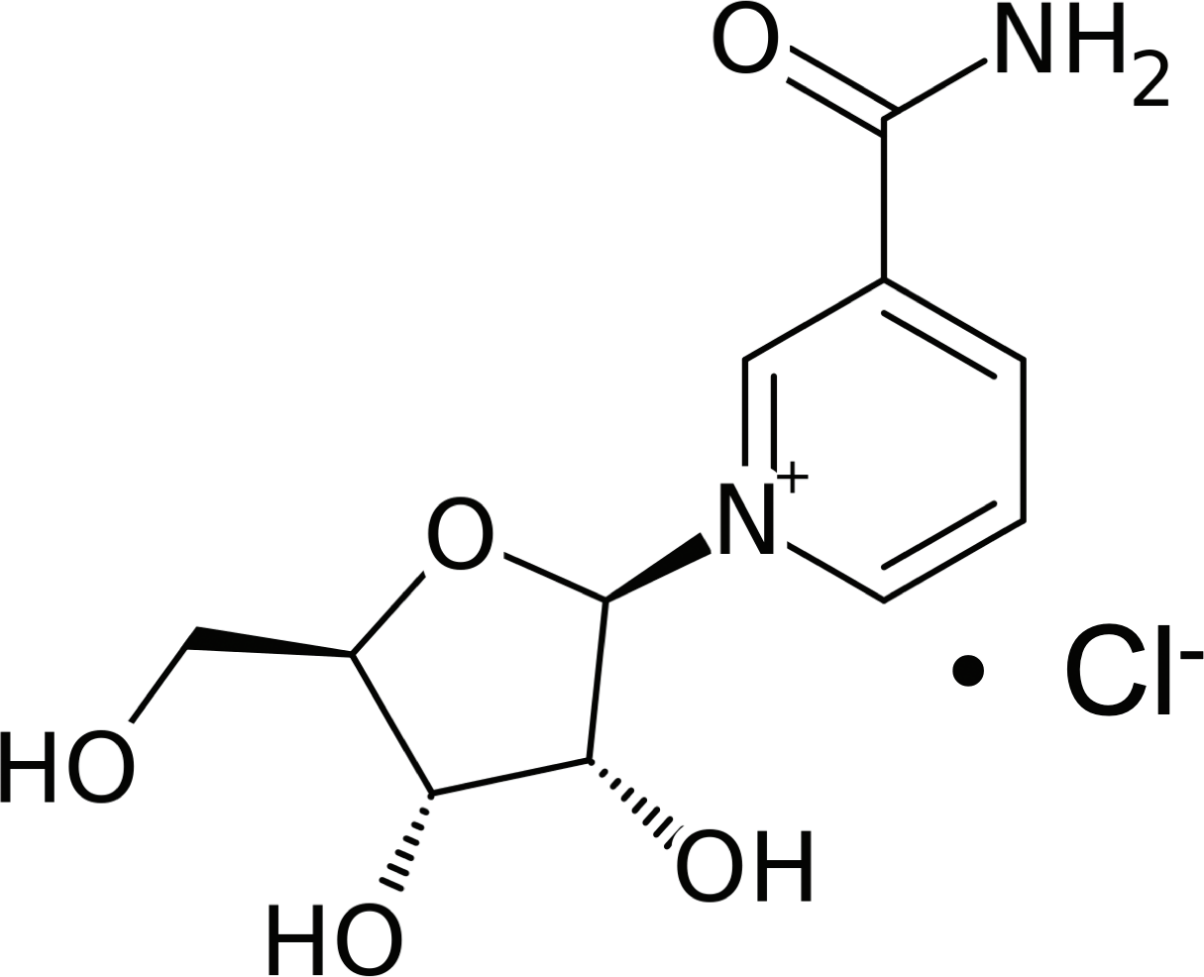
Chemical structure of nicotinamide riboside chloride.

Nicotinamide riboside (NR) (Fig. 1) is a novel vitamin B_3_supplement that has a unique pathway for NAD^+^ synthesis (1) and has been shown to be present in cow milk (2). NR has been proven to increase the available NAD^+^ in mammalian cell lines by up to twofold (3). Over the last decade, NR has been at the centre of a concerted effort to assess its NAD^+^ boosting effect on pathologies with supplementation showing encouraging results in models of Alzheimer’s (4), mitochondrial disease (5), high-fat diet-induced obesity (6), type 2 diabetes (7), noise-induced hearing loss (8), and muscular dystrophy (9). A recent human pharmacokinetics study also indicated a more than twofold increase in human blood NAD^+^ after one single oral dose of NRCl (10) and a published safety assessment states that the lowest adverse effect level of NR is 1,000 mg/kg/day and the no-observed-adverse-effect level (NOAEL) is 300 mg/kg/day (11), making NR completely safe as a nutritional supplement considering its recommended dosing strength of 250 mg daily according to the Niagen™ instructions. The chloride salt of NR (NRCl) is currently marketed in a capsule formulation. However, a full understanding of how different formulation strategies of NRCl may impact upon the efficacy of its delivery is yet to be reported. An appropriate formulation strategy is needed for all supplements, such as NR, in order to optimise their potential health benefits. Physicochemical characterisation of molecules is a vital starting point in product development and optimisation whereby key properties of the molecule are elucidated. These properties include p*K*a, log *P*, solubility, melting point, degradation mechanics, and kinetics. Such properties are the subject of multiple selection and performance criteria for therapeutic candidates such as the rule of five (12) and the biopharmaceutical classification system (BCS) (13).

A full physicochemical characterisation of NR is lacking in the literature. Therefore, this study aims to establish key properties of NRCl to enable the evidence-based development of NR formulations.

## Materials and methods

### Materials

NRCl (Niagen™)was kindly donated by Chromadex^®^ lnc. Nicotinamide (Nam) and D(-)-ribose, sodium phosphate monobasic, sodium phosphate dibasic, sodium acetate, sodium chloride, pepsin, and HPLC grade CHROMASOLV™ acetonitrile were purchased from Sigma-Aldrich Company Ltd. Hydrochloric acid was purchased from VWR Ltd.

### Experimental determination of pKa

NR stock solution was added to nine respective buffer solutions ranging from pH 1.5 to 12.8 and analysed by UV spectroscopic scanning over 200–500 nm using a Cary 50 scan UV-visible spectrophotometer (Agilent Technologies, USA). As recommended in the literature, seven NaOH solutions were prepared for exact determination using a narrower pH range of 11.0–12.2 and the average of these values was used for determination of p*K*a. Equation 1 is a modified Beers–Lambert and Henderson–Hasselbalch equation (14). This was used to calculate p*K*a. *A*_I_ is the absorbance of the ionised species, *A*_M_ is the absorbance of the molecular species, and *A* is the absorbance at any given pH. The same method as above was then repeated using the same parameters and equipment for a duplicate result.

$$\text{p}K_{\text{a}}=\text{pH}+\log\frac{A_{\text{I}}-A}{A-A_{\text{M}}}$$ Equation 1

### Calculation of log P

The log *P* value of NRCl was predicted using the MarvinSketch (ChemAxon Ltd, Hungary) software based on the compound structure. A molecular model and overall log *P* value were generated using the partition log *P* calculation.

### HPLC method development

The HPLC system consisted of an Agilent Infinity 1,220 LC equipped with a variable wavelength UV detector (Agilent Technologies, Germany). A Phenomenex^®^ Synergi™ Hydro-RP column (80 Å, 4.6 × 50 mm, 4 μm particle size) was used for separation and detection of NR and Nam. The mobile phase consisted of a mixture of acetonitrile and phosphate buffer at pH 6. All chromatographic analyses were performed in gradient mode as detailed in Table 1. The column compartment was kept at 25°C and the flow rate was kept constant at 1 mL/min throughout the experiment. The injection volume was 20µL, and the UV detection wavelength was set at 266 nm. This method gives a retention time of 1.00 ± 0.05 min for NR and 2.15 ± 0.05 min for Nam.

**Table 1:** Details of the gradient method used in HPLC analysis

Time (min)	Acetonitrile(%)	Aqueous(%)	Flow (mL/min)
0	2	98	1
1.5	30	70	1
3.5	30	70	1
4	60	40	1
5	60	40	1
6.5	2	98	1

### Aqueous solubility determination

Three pH environments, namely pH 2.0, 5.0, and, 7.4, were used to determine the aqueous solubility of NR. Each buffer was added to a glass vial and stored at 4°C prior to the experiments. NRCl powder was then slowly added over a 24-h-periodunder constant mixing until the solution was clearly saturated (visual observation, *n* = 3). Aliquots were then drawn from each vial and passed through a 0.22 μm syringe filter unit (Millipore, Merck, USA). Samples were then analysed using the validated HPLC method.

### ^1^H nuclear magnetic resonance (NMR) spectroscopy and quantification

A Bruker^®^ Ultrashield 400 MHz NMR was used for NMR spectroscopy. All samples were prepared using deuterated water. For ^1^H NMR spectra acquisition, 256 scans were used. A relative quantitative NMR (qNMR) method was established and validated by comparing the integral values of NR chemical shift peaks to the integral values of the peaks of Nam. The integral of a peak is directly proportional to the number of nuclei the peak represents and the molar concentration of the compound in the sample (14). The percentage of NR was calculated using Equation 2 (modified from the literature), where *I*_NR H2_ and *I*_Nam H2_ were the integral values of the singlet of NR H2 at 9.6 ppm and the singlet for Nam H2 at 8.6 ppm, respectively. *N* represents the number of nuclei (protons) that contribute to each peak. In this case, *N* = 1 for both NR H2 and Nam H2 peaks as they represent one proton each.

$$\%\;\text{NR}\;\text{in}\;\text{smaple}=\frac{\left(I_{\text{NR}\;\text{H2}}/N_{\text{NR}\;\text{H2}}\right)}{\left(\frac{I_{\text{NR}\;\text{H2}}}{N_{\text{NR}\;\text{H2}}}\right)+\left(\frac{I_{\text{Nam}\;\text{H2}}}{N_{\text{Nam}\;\text{H2}}}\right)}\times 100$$ Equation 2

This method was validated by comparing the theoretical percentage of NR and Nam mixtures in five different ratios (*n* = 3) to the percentage mix calculated from Equation 2. The mixtures used were 100% NR, 75% NR: 25% Nam, 50% NR: 50% Nam, 25% NR: 75% Nam, and 100% Nam. A *R*^2^ value of 0.999 was achieved for linearity for this validation.

### Assessment of NRCl stability in simulated gastrointestinal fluids

NRCl powder was tested under simulated GI conditions to assess the stability of the raw material in fluids relevant to the GI physiology. These experiments were carried out using a Copley™ dissolution bath equipped with the USP II paddle apparatus at a stirring rate of 75rpm and a temperature of 37°C± 0.1°C. Five dissolution media were tested (Table 2). 0.1N HCl, USP simulated gastric fluid without pepsin (USP SGF_W/O pepsin_), and USP simulated gastric fluid with pepsin (USP SGF) were used to simulate the stomach environment. Phosphate standard buffer (Int. Pharm. SIF) and simulated intestinal fluid pH 6.8 (USP SIF) were used to simulate the intestinal environment. A 24-h simulation was also performed using the USP prolonged release (USP PR) method whereby for the first 2 h of the experiment the NRCl was added to 750 mL 0.1N HCl. Subsequently, 250 mL of 0.2M trisodium phosphate was added and pH adjusted to pH 6.8 for the remaining 22 h. This step-change pH model simulates the pH change in traversing from the stomach to the intestine (15).

**Table 2:** Media used along with composition, pH, and reference source

Medium	Component	Concentration	pH	Reference
HCl	HCl	0.1N	1.2	–
USP SGF_W/O pepsin_	HCl NaCl	0.1N 2.0g/L	1.2	USP
USP SGF	HCl NaCl Pepsin	0.1N 2.0g/L 3.2g/L	1.2	USP
USP SIF	KH_2_PO_4_ NaOH	6.81g/L 0.896g/L	6.8	USP
Int. Pharm. SIF	KH_2_PO_4_ Na_2_HPO_4_	3.4g/L 3.53g/L	6.8	(30)
USP PR	HCl Na_3_PO_4_	0.1N 76.02g/L	1.2–6.8 after 2 h	USP

### Determination of the mechanism and kinetics of NRCl degradation in aqueous solutions

NRCl degradation products and mechanism were determined and confirmed using ^1^H NMR. NRCl degradation kinetics in aqueous solution was determined using three pH environments: pH 2.0, 5.0, and 7.4 (each with a constant ionic strength of 0.3M adjusted using NaCl), and three experimental temperature conditions, namely, 55°C, 65°C, and 75°C, respectively. In all cases, five replicates were used for pH–temperature combinations and each was an individual, sealed, and labelled glass vial with 20mL of the respective buffer. For each experiment, these vials were left overnight to equilibrate to the respective temperature environment in a temperature-controlled oven. At the start of each experiment, the vials were removed and a stock solution of NR was added. Aliquots were then extracted from each vial at this point for a *t* = 0 and the vials were placed back into the oven until the next time point. The sampling intervals were every 2, 1, and 0.5 h for 55°C, 65°C, and 75°C, respectively, and a total of five time points were used for each individual vial. When an aliquot was removed, it was dispensed into a 2mL micro tube and placed in ice to quench the reaction until preparation for HPLC analysis.

### Thermal analysis of NRCl

Thermal analysis of NR was conducted on a Q100 model single furnace heat flux differential scanning calorimeter (TA Instrument, USA). The instrument was calibrated at the respective ramp rate with indium and zinc for both melting point and the heat of fusion. Dry nitrogen was purged at a flow rate of 50L/min through the sampling chamber to maintain an inert atmosphere. About 3–5mg of the sample was accurately weighed on Perkin Elmer^®^ standard aluminium pans and crimped with aluminium pan lids. The crimped pan set was then subjected to a ramping rate of 10°C/min from 20°C to 200°C.

### Combinatorial use of DSC and 1H NMR to understand NRCl thermal decomposition

DSC was used in conjunction with ^1^H NMR spectroscopy to assess NR stability at certain temperatures and confirm compositional change throughout the entire thermal degradation process. Samples were first run with loosely covered pans to 115, 120, 125, 130 or 140°C respectively then quench cooled to −40°C to acquire thermal profiles comparable to previous runs. This was then repeated in pans with no lids and these pans were then put in 1mL of D_2_O until all the contents were fully dissolved. The resulting D_2_O solutions were then subjected to ^1^H NMR analysis, as previously detailed, to assess the stability of NRCl at each stage of its melting motif.

### Statistical analysis

Statistical analysis was carried out using Graphpad™ Prism 7 software. The particular statistical test used for each set of data is reported in each method respectively. For aqueous solubility analysis, a ONE-WAY ANOVA was used with Tukey’s multiple comparisons. A significance value (*P* value) that was smaller than 0.05 was indicative of a significant difference between the two sets. Linearity and raw data processing were carried out on Microsoft Excel™.

## Results

### pKa determination and log P prediction

**Fig. 2:**
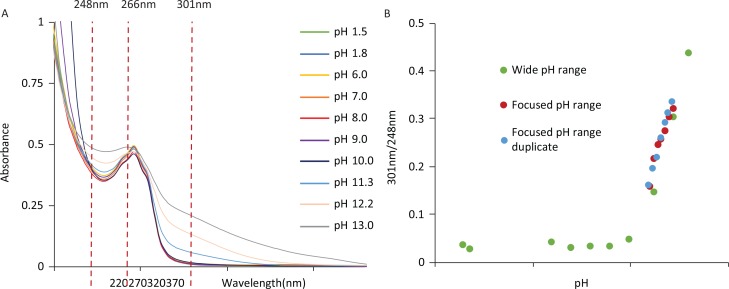
(A) UV spectra of NR at varying pH conditions from pH 1.5 to pH 13. (B) 301 nm/248 nm absorbance against pH for NRCl with preliminary wide pH range (green) to inform for focused experiments (red and blue) to determine pKa values. pKa values of 11.5 ± 0.2 and 11.5 ± 0.1 were obtained from the focused pH range and focused pH duplicate respectively.

Figure 2A shows the UV spectra of NR changes at each of the buffers used. An isosbestic point is observed at the lambda max at 266 nm for all tested pH values. The wavelengths at which most change is noticed are 301 nm and 248 nm, respectively. Figure 2B shows the 301 nm/248 nm absorbance ratio, adjusted to baseline, against pH to show the ionisation profile of NRCl over a wide range of pH values (green) and two focused ranges (red and blue). The p*K*a of NRCl was calculated to be 11.5 ± 0.2 from the first focused experiment based on these experimental results using a modified Beers–Lambert and Henderson–Hasselbalch equation (Equation 1). A duplicate value of 11.5 ± 0.1 was then obtained from a second focused experiment. A predicted log *P* of –6.25 was generated by chemaxon software after inputting the chemical structure of NRCl, suggesting that NRCl is an extremely hydrophilic compound.

### Aqueous solubility

The solubility of NRCl at pH 2.0, 5.0, and 7.4 was measured to be 972.7 ± 8.1, 860.5 ± 31.0, and 826.0 ± 34.4 mg/mL respectively. Albeit showing statistically recognisable pH-dependent solubility towards the lower end of the pH scale, NRCl should be considered freely soluble (100–1,000 mg/mL) in all three tested pH environments according to the USP definitions (16).

### Degradation mechanism in aqueous solutions

**Fig. 3:**
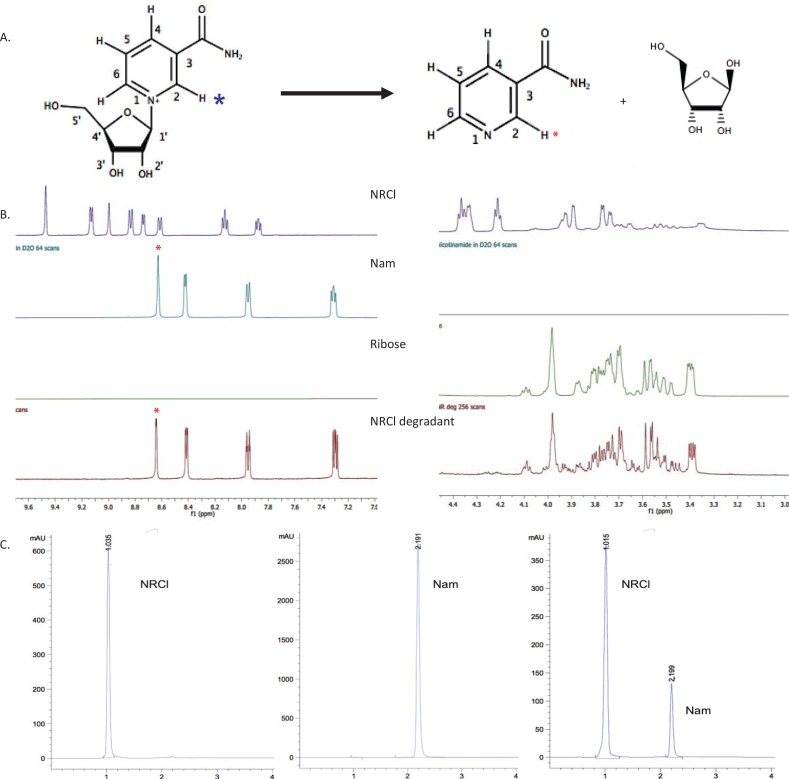
(A) The proposed scheme for degradation of NR to nicotinamide and sugar under basic conditions. (B) Proton NMR spectra of (from top to bottom) NR, Nam, D(-)-ribose, and degraded NR after forced degradation with stars representing the H2 proton on NR and Nam, respectively. (C) HPLC chromatogram using UV detection at 266 nm showing (left) NR with a retention time of 1.015 min, (middle) the reference Nam with a retention time at 2.199 min, and partially degraded NR showing a mixture of NR and Nam.

**Fig. 4:**
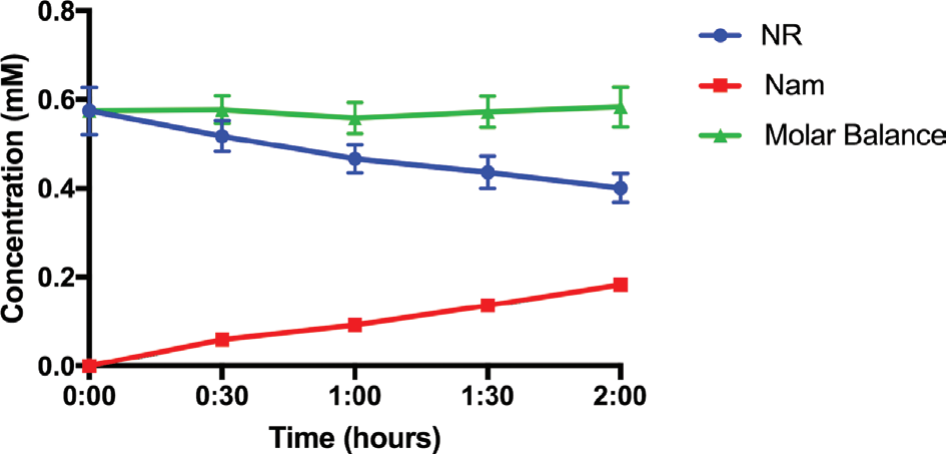
Concentration (mM) of NR (blue), Nam (red), and the total molar sum of both NR and Nam (green) during forced degradation of NR at 120°C. Quantification was performed using the developed HPLC assay. Error bars represent standard deviation of five replicates (n = 5 at each time points).

A forced degradation study was performed by subjecting the NR-deuterated water solution to an elevated temperature condition (75°C) for prolonged periods of time. Samples were taken every 4–6 h and analysed using proton NMR to confirm the status of the degradation process. As NR degrades, nicotinamide and sugar are formed. Figure 3A illustrates the proposed mechanism of NR degradation by base catalysed hydrolysis. Figure 3B shows the proton NMR spectra for NR, Nam, D(-)-ribose, and degraded NR. It can be seen that as the NR peak decreases in intensity, the Nam peak proportionally increases (Fig. 3B, left). It is also evident (Fig. 3B, right) that proton peaks identical to those of the reference ribose were present in the degraded NR product, confirming formation of ribose upon NR degradation. The HPLC chromatograms in Fig. 3C depict the elution of NR and Nam, respectively, from their own standard solutions and, from a sample that contains partially degraded NR. Figure 4 shows the concentration change of NR and Nam over time (quantification by HPLC assay), respectively, compared to the molar balance (NR plus Nam). As NR degrades and decreases in concentration, the concentration of Nam proportionally increases and the molar balance (the sum of both NR and Nam) remains consistent at approximately 0.57 mM.

### Degradation kinetics in aqueous solutions

**Fig. 5:**
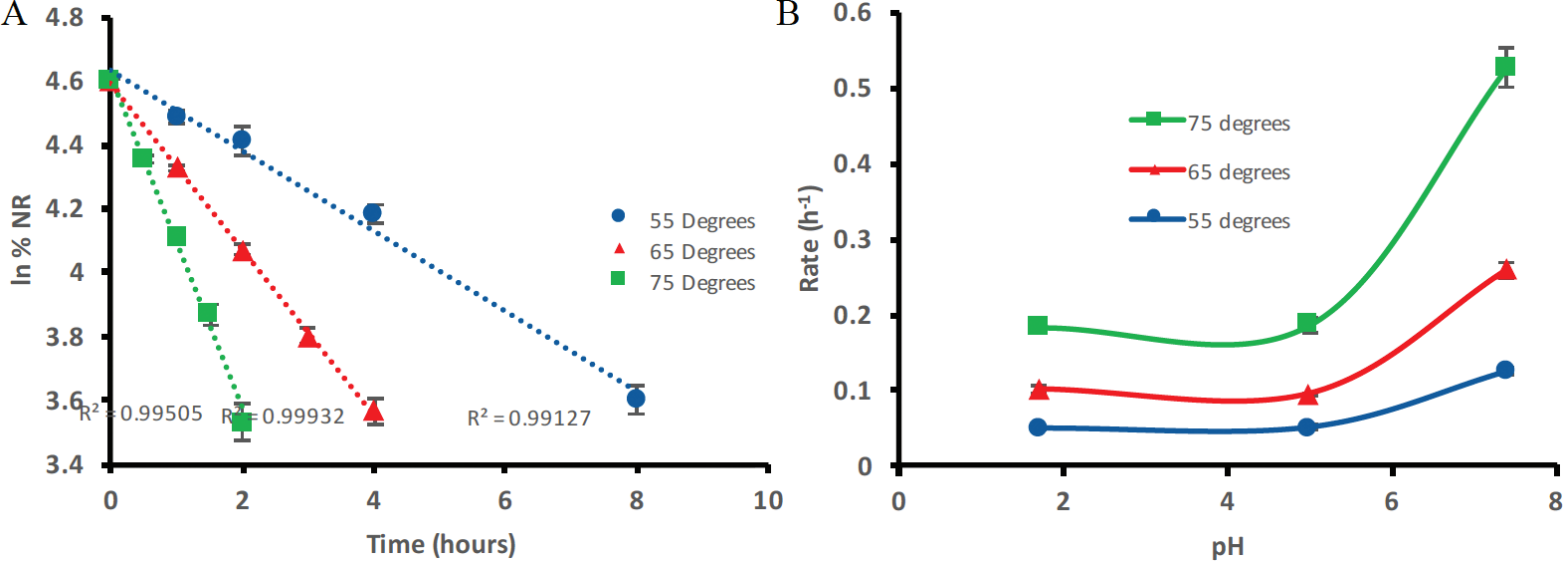
(A) The average (n = 5) natural log first-order kinetic plots of NRCl degradation at 55°C, 65°C, and 75°C, respectively, at pH 7.4. (B) Overlay of the observed rate/pH profiles at 55°C, 65°C, and 75°C, respectively. Exponential relationship can be seen. Error bars represent standard deviation.

NR shows pseudo first-order degradation kinetics (Fig. 5A). Due to the nature of the experiments in differing pH environments, the utilisation of an OH^–^during hydrolysis as described in Fig. 3A, would mean that the reactions are second order. However, the use of buffer systems makes this effect negligible as the pH environment has the capacity to keep the OH^−^/H_3_O^+^ concentration constant and also in great excess compared to NR. As a result, one cannot truly describe these data as first order but rather as ‘pseudo first order’ and the rates themselves shall be referred to as ‘observed rates’ regulated by an ‘observed rate constant’ *k*_obs_. The kinetics of NR degradation in aqueous solutions can be described using Equation 3 where *C* is the concentration of NR at a given time point, *C*_0_ is the concentration of NR at time zero, *t* is the time in minutes, and *k*_obs_ is the observed rate constant.

$$\text{ln}\left[C\right]=\ln\left[C_{0}\right]-k_{\text{obs}}t$$ Equation 3

Good linearity was seen for all pH and temperature environments tested when the natural log of the percentage NRCl remaining was plotted against time with all *R*^2^ values >0.99. From these plots, the average rates were determined (*n* = 5).

Figure 5B shows the effect of pH and temperature on the degradation rate of NR. Generally, the rate of degradation increases as temperature increases, when in the same pH environment. An approximate twofold increase in the degradation rate was calculated for every 10°C temperature elevation. Raising the pH value from 5.0 to 7.4, on the other hand, doubled the degradation rate of NRCl at each respective temperature. The impact of temperature on the rate of a reaction can be described using the Arrhenius equation (Equation 4).

$$k=Ae^{-Ea/RT}$$ Equation 4

Here *k* is the rate of reaction, *T* is the absolute temperature, *A* is the pre-exponential factor, *R* is the universal gas constant, and *E*a is the activation energy.

Table 3 shows the Arrhenius descriptors for the NR degradation rate at pH 2.0, 5.0, and 7.4 respectively. The good linearity (*R*^2^ ≥ 0.99) in all three cases confirms the temperature dependence of NR degradation under all tested pH environments. The activation energy (*E*a) calculated for pH 2.0 is 75.4 (±2.9) kJ/mol compared to a value of 76.9 (±1.1) kJ/mol for pH 5.0. The exponential factor (*A*) values are also similar, 4.8 × 10^10^ per min for pH 2.0 compared to 3.2 × 10^10^ per min for pH 5.0. The *E*a and *A* values obtained under the pH 7.4 condition, on the other hand, show more significant differences. At pH 7.4, the pre-exponential factor *A* is two orders of magnitude higher at a value of 1.8 × 10^12^ per min than that obtained from the two lower pH values. In addition, the activation energy,82.8 (±4.4) kJ/mol for NRCl degradation in a pH 7.4 aqueous solution is higher than that calculated at pH 2.0 and pH 5.0, respectively.

**Table 3:** The Arrhenius linearity equation, R^2^, activation energy (kJ/mol), standard deviation value for each E_a_ (n = 5), and pre-exponential factor (/min)

pH	Ln *k* = f(1/*T*) + ln(*A*)	*R*^2^	*E*a (kJ/mol)	S.D.	*A* (/min)
2.0	–9,064.2(1/T) + 24.587	0.999	75.4	2.9	4.8 × 10^10^
5.0	–8,935.7(1/T) + 24.204	0.990	76.9	1.1	3.2 × 10^10^
7.4	–9,959.1(1/T) + 28.219	0.994	82.8	4.4	1.8 × 10^12^

### NRCl stability in simulated GI fluids

**Fig. 6:**
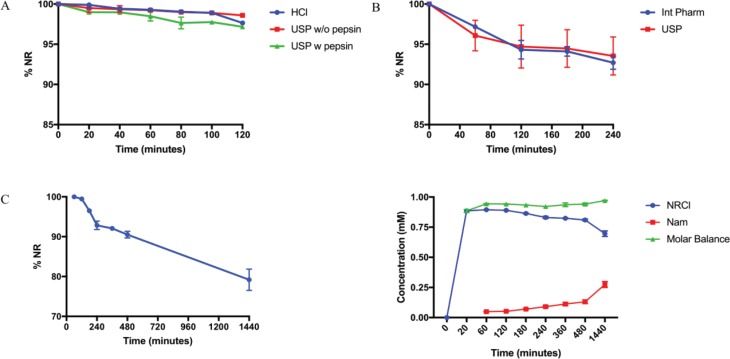
(A) The stability of NRCl in three different gastric simulated fluids over time with %NR describing NRCl stability. Each point represents n = 3 with errors bars for standard deviation. (B) The stability of NRCl in two simulated intestinal fluids over time with %NR describing NRCl stability. Each point represents n = 3 with errors bars for standard deviation. (C) The stability of NRCl in GI simulated fluids over 24 h. (D) The stability of NRCl (blue), occurrence of Nam, and the molar balance between the two species in GI simulated fluids over 24 h shown in molar values (mM).

In the three gastric simulated fluids tested, NRCl displayed very little degradation under physiological temperature and agitation of 75rpm. After 2 h, there was 97.64 ± 0.08%, 98.59 ± 0.10%, and 97.17 ± 0.19% of NRCl remaining in HCl, USPSGF without pepsin, and USPSGF, respectively (Fig. 6A). More pronounced degradation was observed in the simulated intestinal fluids. After 2 h, there was 94.32 ± 1.16% NRCl remaining in intestinal SIF and 94.71 ± 2.66% remaining in USP SIF, respectively. Another 2 h exposure to the same SIFs resulted in 92.71 ± 0.81% and 93.54 ± 2.37% remaining NRCl content in the respective medium (Fig. 6B). In the 24-h GI simulation experiment, there was 90.51 ± 0.82% remaining after 8 h and 79.18 ± 2.68% remaining by the final time point at 24 h (Fig. 6C). By plotting the quantified NRCl (blue) and Nam (red) within the simulated GI fluid, Fig. 6D depicts how, as NR decreases due to degradation, Nam accumulates. The constant molar balance between NRCl and Nam throughout the entire experiment (green, Fig. 6D) also confirms occurrence of only one degradation pathway.

### Solid-state behaviour and decomposition under elevated temperatures

**Fig. 7:**
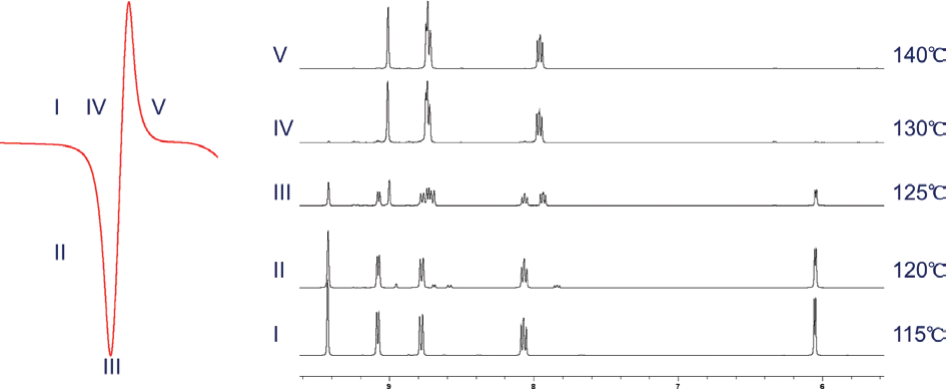
(A) Melting motif of NRCl. I – 115, II– 120, III – 125, IV – 130, and V –140°C. NR shows complete degradation at 130°C on heating. (B) ^1^H NMR spectra from 9.5 to 5.5 ppm of NRCl that was heated to respective temperature then quenched to −40°C. NRCl was completely intact under condition I but was completely degraded under condition IV with increasing levels of degradation seen in conditions II and III.

NRCl shows an onset of melting at 120.7 ± 0.3°C and a peak of melting at 125.2 ± 0.2°C, as indicated by the DSC thermogram, followed by an immediate, sharp exothermic event that peaks at 130.8 ± 0.3°C (Fig. 7A). Due to such uncommon motif, additional experiments were conducted to further assess this exothermic event. Critical temperatures were identified for analysis. These temperatures corresponded to; slightly prior to occurrence of any thermal events, at the onset and at the peak of the melting at 115°C, 120°C, and 125°C respectively, as well as the peak exotherm and the end set of the immediate exotherm at 130°C and 140°C, respectively. Figure 7B shows the ^1^H NMR spectra of NRCl samples that were heated to the abovementioned five critical temperatures, respectively, followed by immediate quench-cooling to –40°C and dissolution in deuterium dioxide for spectral analysis. Using the qNMR method, it can be seen that NR shows only 2% degradation at 115°C, which is to be expected as this is prior to the thermal events. However, at 120°C a 7% degradation is seen and this continues to increase to 55 and 98% degradation at 125°C and 130°C respectively.  At 140°C, only 0.45% of the sample was NR.

## Discussion

NR is a nutrient that has been shown to have significant therapeutic potential and has undergone multiple investigations to assess the effect of its supplementation into models of disease (4–9). Physiologically, there are a wide range of pH environments that any nutrient, especially the ones administered orally, may be exposed to. Even an intravenous injection of NR would be exposed to pH 7.4 in the blood, let alone the more complex pH environment through the GI tract. However, there are currently no stability studies conducted on NRCl in the literature. The aim of this study was, therefore, to characterise NRCl and determine its physicochemical characteristics, in particular its stability, and behaviour in order to inform for advanced formulation of this promising nutritional supplement. The data acquired will also inform about conditions that may affect processing, production, and storage.

The fact that the main degradation product of NRCl is Nam, a vital vitamin that is used on the same biochemical pathways as the parent NR, provides a significant hurdle to overcome when attempting to deliver NRCl as a nutritional supplement. While this may, at first, appear to be negligible due to the final product of both NR and Nam utilisation being NAD, Nam may actually counter or antagonise some positive effects known to be attributable to NRCl supplementation. For example, Nam is a known Sirtuin (SIRT) inhibitor (17–19), whereas supplementation of NRCl provides increased amount of substrate (NAD^+^) to SIRTs (5). Therefore, if during the bio-distribution phase of NR, there is breakdown to Nam and sugar, Nam could inhibit SIRT enzymes, leading to a reduced effect of the therapy and could lead to the possibility of false negatives or other erroneous results in studies. Therefore, due to the possible impact that Nam could have on any potential benefit from NR supplementation, understanding the conditions in which NR is unstable is of the upmost importance.

Although different simulated GI media have been extensively used for dissolution testing (20, 21), they are rarely used to predict a compound’s stability on GI transit. While there appears to be negligible degradation of NRCl in simulated gastric media, this was to be expected according to the predicted degradation scheme. However, there appears to be significant degradation in simulated intestinal media due to the increased pH of these simulated environments. Furthermore, this point was reinforced by the 24-h simulation of the GI where only 71.69 (±1.92)% of NRCl remained at the end of the experiment. This observation underlines the susceptibility of NRCl to degradation in neutral pH environments. The data suggest that the stability of NRCl in all three of the simulated gastric fluids was similar. The same can also be said of the two simulated intestinal fluids, where there was no difference in degradation between the Int. Pharm. and USP fluids. While this model does not take certain factors such as molecules being adsorbed into consideration, it does indicate that there is a significant barrier to the efficient oral delivery of NRCl.

Due to the results obtained during the in vitro GI transit simulations, further studies to investigate the effect of pH and temperature were carried out. pH has a significant influence on NRCl degradation. Neutral conditions, such as pH 7.4 used in one aspect of this work, appear to have a significant effect on the degradation of NR in a similar trend seen during the in vitro GI simulation above. In the Arrhenius model of NR degradation, the observed rate of degradation of NR is higher at pH 7.4 compared to pH 2 and pH 5. These findings support the hypothesis that the degradation of NR will be related to the proportion of OH^−^ ions in the solution and that NR is degraded by base-catalysed hydrolysis. Temperature has a predictable effect on NRCl degradation where an increase of 10°C under any pH condition approximately doubles the *k*_obs_. As the degradation mechanism for NRCl in aqueous solution is hydrolysis, researchers must take care when using aqueous solutions of NRCl, particularly at elevated pH values where the excessive OH^−^ can promote such hydrolytic destruction. It is hypothesised that there is a slightly different degradation mechanism for NR under acidic conditions compared to degradation under neutral or basic conditions. This would explain the observed base-catalysed hydrolysis and a difference in *E*a values between acidic and neutral pH values. The Arrhenius model can be used as a rough guide to understand the stability of NRCl in buffered solutions at pH 2.0, 5.0, and 7.4. However, it would be advisable for researchers to confirm the NRCl and Nam content prior to and following any experiments to preclude significant breakdown.

Charge, molecular weight, and lipophilicity are key descriptors to predict permeability (12). Compounds with estimated log *P* values lower than 1.72 are considered to display low permeability (22). Physiologically, NRCl should be Class III according to the BCS due to its high solubility but poor predicted permeability, with a predicted log *P* of −6.25 (13). This informs for further investigation to assess the mechanism by which NRCl can pass through biological membranes. One would also predict that if NRCl displayed poor permeability, an immediate release formulation may result in a large amount of excreted compound and from the data gathered from the simulated GI fluids, a large proportion of nicotinamide could be formed and absorbed. This again reaffirms the concern stated earlier that an active degradation product with potential effects antagonistic to that of NR supplementation could accumulate in large concentrations after administration due to being exposed to a hostile environment.

The ^1^H NMR spectra suggested that the degradation products of NRCl in aqueous solutions are Nam and sugar. The molar balance experiment adds further evidence to the proposed degradation mechanism hypothesis. Namely, that for every molecule of NR that degrades, one molecule of Nam and one of sugar, respectively, are formed. This is important as the nicotinamide concentration can be used as a marker for NR degradation either alone or in tandem with NR concentration. Indeed, one of the advantages of the developed HPLC method is that it is able to separate and quantify NR peaks and Nam peaks with a separation resolution of 13.9, which greatly exceeds the acceptable resolution recommended by the FDA (>2) (23). This method provides fast detection of NR and its degradation product, Nam. It is also important to note that hydrolytic splitting off of Nam from NAD^+^ has long been reported. The NAD^+^ hydrolytic degradation is principally catalysed by enzymes in the particulate fractions of the intestinal homogenates and may follow a number of different pathways to form different degradants. The reported hydrolytic mechanisms for NAD^+^ include formation of Nam through hydrolysis of the ribosyl–pyridinium bond and that of nicotinamide mononucleotide (NMN) through breaking of the pyrophosphate bond. It is also evident in the literature that environmental pH plays a significant role in NAD^+^ hydrolysis kinetics as Nam is the principle degradant between pH 5.0 and pH 6.0, whilst NMN becomes the more favoured product when above pH 7.0. It is interesting as NMN is subsequently dephosphorylated to form NR, which according to our discovery should then undergo further hydrolysis when pH > 7.0 to produce Nam; yet in Baum’s work, the release of NMN seems to have preceded the cleavage of Nam from NR in the reported test environment (brush border membrane in rat intestine) (24). Although further understanding of facilitators and obstructers for NR degradation in physiological environments where fluids have elevated pH values (i.e. the distal region of small intestine, blood) is yet to be established, the current study provides the physicochemical basis of such degradation processes.

The conclusion that one mole of NR breaks down to one mole of Nam enabled the development of a relative qNMR method for quantitative analysis of the NR and Nam content change throughout the entire thermal degradation process. This method allowed clarification of the sequential endothermic–exothermic events noted on the DSC thermograms. Such a particular sequence of thermal events is not obviously distinguishable as an exothermic heat flow can represent crystallization (25), polymorphic change (26), or oxidation (27). Interestingly, a similar pattern of thermal events was also observed during the heating of furosemide co-crystals (28). The authors have attributed this sequential event to the melting and immediate degradation of furosemide upon reaching the peak of melting based on previous studies investigating furosemide decomposition (29). The combinatorial use of DSC and proton NMR confirmed that the observed exothermic events immediately post the peak of NRCl melting in our study were the result of NRCl thermal decomposition. Quantitative analysis using the qNMR method helped elucidate the exact extent of decomposition (onset to completion) at each critical temperature along the journey. Such data provide, for the very first time in the current literature, an explicit upper-temperature limit for processing of NRCl and its products which can impact the production of supplements at multiple stages.

The integrity of the NR molecule must be at the forefront of all development for NR products. Firstly, this includes reducing, as much as possible, the degradation of NR during processing, packaging, transport, and storage. Secondly, ensuring that when NR is administered it does not degrade to Nam and sugar, due to the potential physiological effects and environments discussed above.

## Conclusion

NR has significant potential as a nutritional supplement. However, here we have reported that NR is a labile molecule with defined and absolute upper limits for processing and handling. An active effort must be made to preclude breakdown of the NR molecule during the development of new NR products and upon its delivery for supplementation. NRCl is predicted to not be stable in the GI tract, post stomach transit. The key physicochemical properties that must be considered when researchers work with NRCl have been described. An HPLC method was developed for the clear distinction of NR from its degradation product Nam. Using this method, we have shown that there is significant NRCl breakdown when exposed to simulated intestinal environments. A novel combinatorial use of ^1^H NMR and DSC was also described, for the first time in the literature, to help understand the thermal profile of NRCl. The optimisation of NR delivery, as with all supplements, will significantly impact on its efficacy. Here, we have described a mechanism by which NRCl degrades to form a possibly antagonistic product when exposed to simulated GI fluids *in vitro*. Further development of NRCl delivery will enable further high-impact research into its promising therapeutic potential. The preceding data are very much the first step *en route* to this goal of supplement dosage for optimisation and provide previously lacking stability data for NRCl.
